# Quality-Controlled Small-Scale Production of a Well-Defined Bacteriophage Cocktail for Use in Human Clinical Trials

**DOI:** 10.1371/journal.pone.0004944

**Published:** 2009-03-20

**Authors:** Maya Merabishvili, Jean-Paul Pirnay, Gilbert Verbeken, Nina Chanishvili, Marina Tediashvili, Nino Lashkhi, Thea Glonti, Victor Krylov, Jan Mast, Luc Van Parys, Rob Lavigne, Guido Volckaert, Wesley Mattheus, Gunther Verween, Peter De Corte, Thomas Rose, Serge Jennes, Martin Zizi, Daniel De Vos, Mario Vaneechoutte

**Affiliations:** 1 Eliava Institute of Bacteriophage, Microbiology and Virology (EIBMV), Tbilisi, Georgia; 2 Laboratory for Molecular and Cellular Technology (LabMCT), Burn Centre, Queen Astrid Military Hospital, Bruynstraat, Neder-over-Heembeek, Brussels, Belgium; 3 Laboratory of Bacteriophage Genetics, State Institute for Genetics and Selection of Industrial Microorganisms (SIGSIM), Moscow, Russia; 4 Unit Electron Microscopy, Veterinary and Agricultural Research Centre (VAR), Ukkel, Brussels, Belgium; 5 Section Health of the Division Well-Being (Belgian Defence Staff), Queen Astrid Military Hospital, Neder-over-Heembeek, Brussels, Belgium; 6 Laboratory of Gene Technology (LoGT), Katholieke Universiteit Leuven, Leuven, Belgium; 7 Department of Physiology (FYSP), Vrije Universiteit Brussel, Jette Brussels, Belgium; 8 Laboratory of Bacteriology Research (LBR), Ghent University Hospital, Ghent, Belgium; University of California Merced, United States of America

## Abstract

We describe the small-scale, laboratory-based, production and quality control of a cocktail, consisting of exclusively lytic bacteriophages, designed for the treatment of *Pseudomonas aeruginosa* and *Staphylococcus aureus* infections in burn wound patients. Based on succesive selection rounds three bacteriophages were retained from an initial pool of 82 *P. aeruginosa* and 8 *S. aureus* bacteriophages, specific for prevalent *P. aeruginosa* and *S. aureus* strains in the Burn Centre of the Queen Astrid Military Hospital in Brussels, Belgium. This cocktail, consisting of *P. aeruginosa* phages 14/1 (*Myoviridae*) and PNM (*Podoviridae*) and *S. aureus* phage ISP (*Myoviridae*) was produced and purified of endotoxin. Quality control included Stability (shelf life), determination of pyrogenicity, sterility and cytotoxicity, confirmation of the absence of temperate bacteriophages and transmission electron microscopy-based confirmation of the presence of the expected virion morphologic particles as well as of their specific interaction with the target bacteria. Bacteriophage genome and proteome analysis confirmed the lytic nature of the bacteriophages, the absence of toxin-coding genes and showed that the selected phages 14/1, PNM and ISP are close relatives of respectively F8, φKMV and phage G1. The bacteriophage cocktail is currently being evaluated in a pilot clinical study cleared by a leading Medical Ethical Committee.

## Introduction

In burn wound care, bacterial infection remains a major therapeutic problem and renders large numbers of thermal injuries virtually untreatable. Whereas *Staphylococcus aureus* remains a common cause of early burn wound infection, *Pseudomonas aeruginosa* is known as the most common and lethal infectious agent in burn centres, essentially due to its intrinsic and acquired resistance to antibiotics [1]–[3].

To improve burn wound patient care, research at the Laboratory for Molecular and Cellular Technology (LabMCT) of the Burn Centre of the Queen Astrid Military Hospital in Neder-over-Heembeek (Brussels), Belgium is investigating alternatives for the treatment of infections with multidrug resistant (MDR) infectious agents, like (bacterio)phage therapy. The use of bacteriophages against bacterial pathogens was first proposed by d'Hérelle in 1917 [4] and has a long and convoluted history. Currently, phage therapy is the subject of renewed interest, as a consequence of the continuing increase in antibiotic resistance worldwide [5], illustrated by the growing number of scientific papers and text books [6]–[12].

However, major obstacles for the clinical application of bacteriophages are the perception of viruses as ‘enemies of life’ [13], the lack of a specific frame for phage therapy in the current Medicinal Product Regulation [14] and the absence of well-defined and safe bacteriophage preparations.

To evaluate the safety and efficacy of bacteriophages in the treatment of burn wound infections in a controlled clinical trial, we prepared a highly purified and fully defined bacteriophage cocktail (BFC-1), active against the *P. aeruginosa* and the *S. aureus* strains actually circulating in the Burn Centre of the Queen Astrid Military.

To our knowledge the present paper describes for the first time, in detail - from the initial bacteriophage isolation to the final composition - a laboratory-based production of a well-defined bacteriophage cocktail.

## Methods

**Figure 1:**
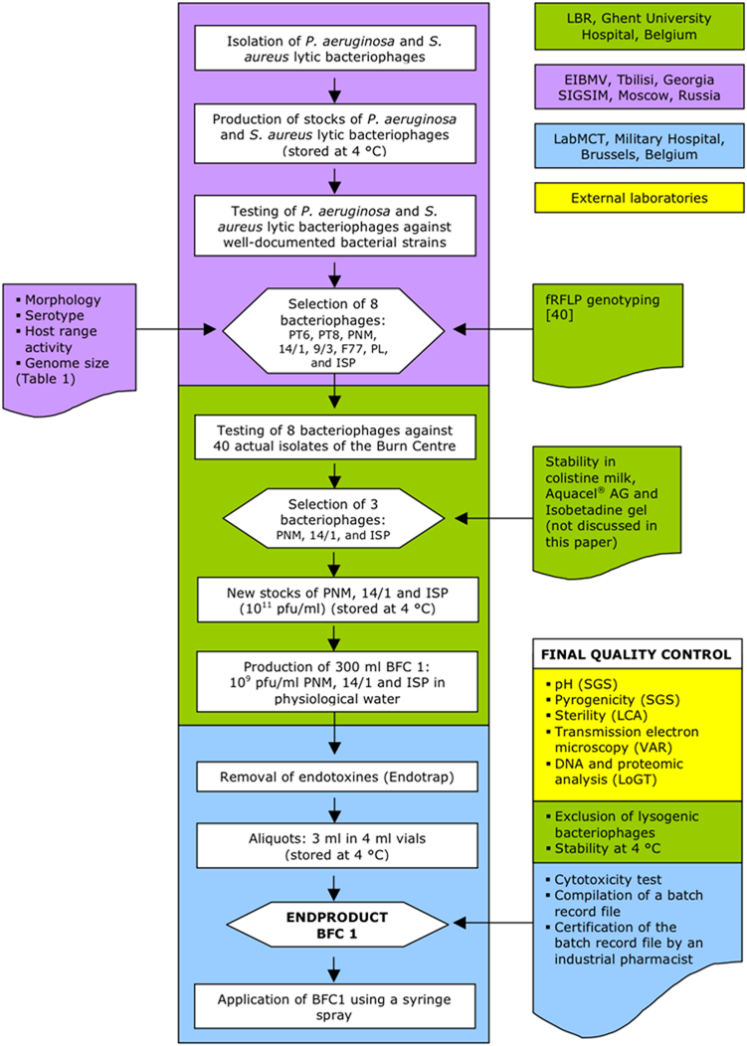
Flow chart of the BFC-1 production and final quality control.

A flow chart of the entire BFC-1 production process and quality control tests is depicted in **Figure 1**.

### Titration of bacteriophage suspensions using the agar overlay method

The bacteriophage titre was determined by assaying decinormal serial dilutions (log(0) to log(−12)) of the bacteriophage suspensions with the agar overlay method [27], [28]. One ml of each dilution was mixed with 2.5 ml molten (45°C) Luria Bertani (LB) (Becton Dickinson, Erembodegem, Belgium), containing 0.7% top agar (Bacto agar, Becton Dickinson), and a suspension of bacteriophage sensitive bacteria (end concentration of 10^8^ cfu/ml) in sterile 14 ml tubes (Falcon, Becton Dickinson). This mixture was plated in triplicate onto 90 mm diameter Petri dishes (Plastiques Gosselin, Menen, Belgium) filled with a bottom layer of 1.5% LB agar and incubated for 18–24 h at 37°C. To estimate the original bacteriophage concentration, plates with one to 100 distinguishable homogenous plaques were counted depending on the phage plaque size. The mean was then calculated for the triplicate plates.

### Initial isolation, separation and purification of *P. aeruginosa* and *S. aureus* lytic bacteriophages

The bacteriophage sensitive strains used during the production and quality control of BFC-1 are *P. aeruginosa* strain ‘573’, were isolated at the Eliava Institute of Bacteriophage, Microbiology and Virology (EIBMV) in the 1970s from bone marrow interstitial fluid, and *S. aureus* strain '13 S44 S′, isolated at the Brussels Burn Centre in 2006 from a burn wound. Initially, *S. aureus* strain Wood 60 (EIBMV collection) was used for propagation of phage ISP, but for the production of this cocktail the phage was propagated on *S. aureus* 13 S44 S. The absence of temperate phages from the host strains was tested as described in a separate section of this paper.

For bacteriophage isolation from natural samples such as sewage and river water, one millilitre of 10×concentrated LB Broth (Becton Dickinson), 1 ml ‘host bacteria’ suspension, containing 10^8^ cfu in LB broth and 9 ml sewage or river water were mixed in a 14 ml sterile tube. This tube was incubated at 37°C for 1.5–2 h. Subsequently, 200 µl of chloroform (Sigma-Aldrich, Bornem, Belgium) was added and the tube was further incubated at 4°C for 1 h. The lysate was aspirated with a sterile 5 ml syringe and passed through a 0.45 µm membrane filter (Minisart, Sartorius, Vilvoorde, Belgium). Bacteriophages were titrated using the agar overlay method, as described above. All plaques with different morphology were touched with a sterile pipette tip, inoculated into 2 ml of sterile LB broth in 14 ml sterile tubes and incubated at 37°C for 2 h. Subsequently, 50 µl of chloroform was added and the tube(s) were incubated at 4°C for 1 h. For each tube, a dilution series (log(0)−log(−4)) was made in sterile 14 ml tubes filled with LB broth. Each dilution was titrated using the agar overlay method. Plates showing 1–10 plaques were analysed in detail. Again, all plaques with different morphology were touched with a sterile pipette tip, inoculated into 2 ml of sterile LB broth in 14 ml sterile tubes and incubated at 37°C for 2 h. This complete cycle was repeated until one plaque morphotype was obtained (homogeneous plaques).

In the case of bacteriophage ISP, which was isolated in the 1920s, porcelain, rather than membrane filters were employed.

### Production of bacteriophage stocks

Bacteriophage stocks were prepared using the double-agar overlay method with minor modifications. One millilitre of lysate (see above) containing 10^3^–10^5^ plaque forming units (pfu) of bacteriophages was mixed with 2.5 ml molten (45°C) Select Alternative Protein Source (APS) LB (Becton Dickinson, Erembodegem, Belgium) top agar (0.7%) and a bacteriophage sensitive bacterial suspension (end concentration of 10^8^ cfu/ml) in a sterile 14 ml tube. This mixture was plated onto ten 90 mm diameter Petri dishes filled with a bottom layer of 1.5% APS LB agar and incubated at 37°C for 16–18 h. Subsequently, 200 µl of chloroform was added to the lids of the Petri dishes and further incubated at 4°C for 1 h. The top agar layer was scraped off using a sterile Drigalski spatula (L-shaped rod) and transferred to a sterile 14 ml tube. The mixture was centrifuged for 20 min at 6 000 g. The supernatant was aspirated using a sterile 10 ml syringe (BD Plastipak, Becton Dickinson) with a 30 G sterile needle (BD microlance 3, Becton Dickinson) and passed through a 0.45 µm membrane filter.

### Selection of therapeutic bacteriophages

Large 24.5 cm square Petri dishes (Nunc, Wiesbaden, Germany) with 2% LB agar were inoculated with the target bacteria (10^8^ cfu/ml LB broth). Each target bacterium was applied in one horizontal strip. As a consequence, the dish contained multiple parallel inoculation strips. Each strip was air-dried and spotted with 5 µl of 10^7^ pfu/ml of each of the bacteriophage suspensions under consideration. The dish was incubated for 16–18 h at 37°C. The obtained lysis zones were evaluated and scored as cl (confluent lysis), ol (opaque lysis), scl (semi-confluent lysis), sp (several plaques) and – (negative reaction).

### Composition and endotoxin purification of the bacteriophage cocktail

Each of the three bacteriophage stock suspensions (PNM, 14/1 and ISP) of the final cocktail was diluted into 100 ml of a sterile 0.9% NaCl solution (B. Braun, Diegem, Belgium) to a final concentration of 3.10^9^ pfu/ml. Subsequently, the three bacteriophage suspensions were mixed in a sterile 500 ml PETG Nalgene® bottle (Nalge Europe, Neerijse, Belgium) to obtain a 300 ml volume of the bacteriophage cocktail (named BFC-1), which contained each bacteriophage at a concentration of 10^9^ pfu/ml. BFC-1 was subsequently purified from endotoxins using a commercially available kit (EndoTrap® Blue, Cambrex BioScience, Verviers, Belgium), according to the instructions of the manufacturer. One column was utilised per 50 ml of BFC-1. Endotoxin purified BFC-1 was collected into a sterile 500 ml PETG Nalgene® bottle and aliquoted into 3 ml doses in sterile 4 ml vials (Brand, Wërtheim, Germany). The final titre of each phage was approximately 1.10^9^ pfu/ml.

### Final quality control

#### pH

The pH of BFC-1 was determined by an accredited laboratory (SGS Lab Simon AS, Brussels, Belgium) in accordance to the European Pharmacopoeia standards (EP6).

### Pyrogenicity

Pyrogenicity was tested by an accredited laboratory (SGS Lab Simon SA) in accordance with the European Pharmacopoeia standards (EP6). A sample of 1.2 ml BFC-1 was intravenously injected in three rabbits. The calculation of the rabbit injection volume in order to achieve the maximum safety level was done according to the following equation: rabbit injection volume = human injection volume x safety factor x rabbit weight/70, with human injection volume = 3 ml - whereby 3 ml is the maximal volume topically applied in the clinical trial and immediate and complete resorption is assumed, to achieve maximal safety; with safety factor = 8 (i.e. maximum safety level) and with rabbit weight = 3.5 kg.

### Sterility

Ten percent of the BFC-1 production was tested for sterility by an accredited laboratory (Laboratoire de Contrôle et d'Analyses, Brussels, Belgium) using the membrane filtration method followed by two weeks of incubation at 37°C, in accordance to the European Pharmacopoeia (EP6).

### Transmission electron microscopy

Bacteriophage particles, with and without target bacteria were analysed by transmission electron microscopy as described by Imberechts et al. [29]. Briefly, suspensions were brought on carbon and pioloform-coated grids (Agar Scientific, Stansted, UK), washed with water and negatively stained with 2% uranyl acetate (Agar Scientific) in water and analyzed using a Technai Spirit transmission electron microscope (FEI, Eindhoven, The Netherlands) operating at 120 kV. Micrographs were recorded using a bottom-mounted digital camera (Eagle, 4X4K, FEI).

### Exclusion of temperate bacteriophages from the host strains

To confirm the absence of temperate bacteriophages, originating from the bacterial hosts used to grow the three lytic bacteriophages, a standard technique for bacteriophage induction using the DNA-damaging antimicrobial agent mitomycin C was carried out, as described by Miller [30].

The host strains used in BFC-1 production (*P. aeruginosa* strain 573 and *S. aureus* strain 13 S44 S, as well as the *P. aeruginosa* reference strain ‘PAO1’) were grown in APS LB broth at 37°C until the early exponential growth phase. Bacterial cultures were aliquoted in 1 ml volumes in sterile eppendorf tubes, covered with aluminium foil thus protecting the bacteria from photoreactivation of drug-induced DNA damage. Mitomycin C (Sigma-Aldrich) was added to final concentrations of 1 or 5 µg/ml [30]. A control tube without mitomycin C was added to evaluate the presence of ‘non-drug-induced’ bacteriophages. The tubes were incubated for 3 h at 37°C. Subsequently, twenty µl of chloroform was added to the control tubes to lyse the bacteria. The lysates were centrifuged in order to separate the intact bacterial cells from the supernatant. The final titre of bacteriophages in the supernatant was determined using the double agar overlay method, as described above.

### Genome sequencing and proteomic analysis

DNA from bacteriophages was isolated using a commercially available kit (Lambda Mini Kit, Qiagen, Hilden, Germany). Purified DNA from bacteriophages 14/1, PNM and ISP was sonicated for 1 s at 20% intensity using a Sonics Vibracell and separated on a 1% agarose gel (Eurogentec, Seraing, Belgium). Fragments between 1000 and 2000 bp were excised from the gel (Qiagen, Venlo, The Netherlands), end repaired using Klenow - T4 polymerase mixture (Fermentas, St.-Leon-Rot, Germany) and phosphorylated using T4 polynucleotide kinase (Roche, Vilvoorde, Belgium). Subsequently, fragments were ligated using T4 ligase (Fermentas) into *Sma*I-linearised pUC19 plasmids and transformed to *Escherichia coli* XL1 blue MRF. This resulted in over 10 000 positive clones after blue white screening for each bacteriophage, of which more than 90% contained inserts between 1 and 2 kb. Plasmids from individual clones were isolated and sequenced using the standard M13f vector primer. After standard ethanol precipitation, samples were separated and analyzed on an ABI 3130 capillary sequencing device (Applied Biosystems, Lennik, Belgium). To complete the genomes (sequenced from each strand for each position), standard primer walking was used. Sequence assembly into contigs was performed using Sequencher 4.1 software (Gene Codes Corporation, Ann Arbor, USA). ORF predictions were made using comparative genomics approaches (tBLASTx), searching for conserved gene products between closely related phages (14-1 vs F8, PNM vs phiKMV and ISP vs G1). To scan the genomes for known toxins/lysogeny-related genes, sequence similarity searches were performed against the non-redundant nr NCBI database.

### Cytotoxicity towards keratinocytes

The effect of BFC-1 on the proliferation of primary neonatal human foreskin keratinocytes was evaluated in triplicate, using the trypan blue dye exclusion test. Keratinocytes (nFS02-006, passage 8) were seeded at a concentration of 2500 keratinocytes per cm^2^ in 24 ml Epilife™ basal growth medium (Cascade Biologics, Invitrogen, Merelbeke, Belgium) in six 75 cm^2^ cell culture flasks (Falcon, Becton Dickinson). One ml of BFC-1 was added to three flasks and 1 ml of sterile physiologic water was added to the three remaining flasks as a control. Flasks were incubated at 37°C, 5% CO_2_ and a relative humidity (RH) of 95% for 5 days without medium change. Three ml of 0.025% trypsin in 0.01% EDTA solution (Lonza, Verviers, Belgium) was added to the flasks. After incubation at 37°C in 5% CO_2_ and 95% RH for 4 min, three millilitres of 0.025% trypsin inhibitor (Sigma-Aldrich) was added and mixed by pipetting. The keratinocyte suspension was collected in a 15 ml tube (Falcon, Becton Dickinson) and centrifuged at 170 g for 10 min. The pellet was resuspended in 5 ml Epilife™ basal growth medium. Subsequently, 100 µl of cell suspension was mixed with 100 µl of a trypan blue staining solution (Biochrom AG, Berlin, Germany) and the living (colourless) and dead (blue) cells were counted using a Bürker cell counting chamber and an inverted microscope (TMS-F, Nikon, Belgium). The mean ratio of dead keratinocytes versus the total number of keratinocytes, expressed in %, compared to the control group, was taken as a measure for the cytotoxicity of BFC-1.

### Stability at 4°C

The stability of a dedicated batch of BFC-1 was monitored on a monthly basis, by determining the titre of each of the three bacteriophages after storage at 4°C. Taking into account test result deviations, inherent to bacteriological methods, titres within a range of 1.10^8^ to 1.10^10^ pfu/ml confer retention of activity and thus stability.

### Stability after spraying

One ml of BFC-1 was aspired in a 2 ml syringe. A spraying head with pore size of 300 µm was fixed onto the syringe and the total volume of cocktail in the syringe was sprayed into a 50-ml tube. Phage titres were determined prior to and after spraying by the double agar overlay method.

### Batch record file

A batch record file was compiled and checked for conformity to the product information file by an industrial pharmacist. Principal aspects contained in the batch record file include:

- the positive advice of the leading ethical committee that approved the BFC-1 clinical study;
- the BFC-1 product information file, which describes, in detail, the production process and the characteristics of BFC-1;
- the material transfer and confidentiality disclosure agreement signed by all partners;
- the filled out working instructions describing all BFC-1 production steps;
- the quality and analysis certificates of all products, materials and equipment (e.g. bench flow), used in the production of BFC-1;
- the results and analysis certificates of all final quality control tests; and
- the labels of the BFC-1 vials.

### Clinical application

**Figure 2:**
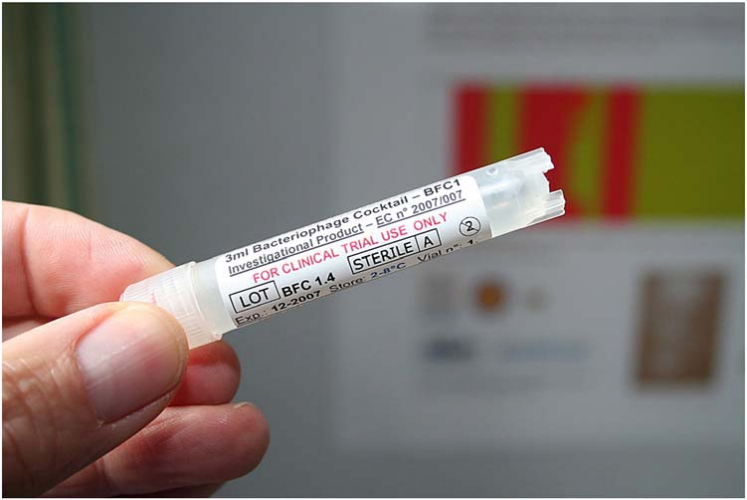
The final product, a defined bacteriophage cocktail.

**Figure 3:**
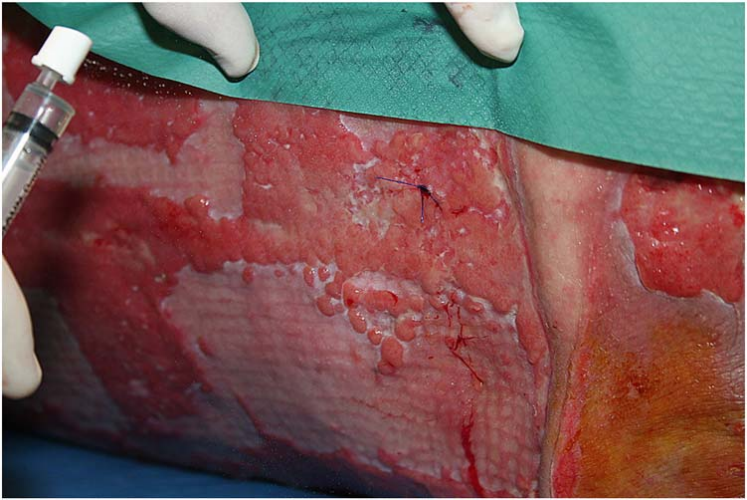
Application of BFC-1 on an infected burn wound using a syringe spray.

Prior to patient application, 3 ml of BFC-1 was aspirated from a ‘single use only’ vial (**Figure 2**) using a sterile 5 ml syringe (B. Braun) with a sterile 20 G×2″ needle (Terumo Europe, Leuven, Belgium). The needle was replaced by a sterile spray nozzle (actuator V04.1313 BC/NR with micromist insert V06.203, Robertpack Engineering B.V., Zwolle, The Netherlands). BFC-1 was sprayed on the infected burn wound (**Figure 3**).

## Results

### Selection and host range activity of therapeutic bacteriophages

The activity of 82 *P. aeruginosa* and 8 *S. aureus* bacteriophages from the collections of the Eliava Institute for Bacteriophage, Microbiology and Virology (EIBMV), Tbilisi, Georgia and the State Institute of Genetics and Selection of Industrial Micro-organisms (SIGSIM), Moscow, Russia was determined against a total of 113 *P. aeruginosa* and 99 *S. aureus* strains, isolated from different clinical and environmental habitats across the world. The complete data on used strains and phages is shown in **Table S1**.

Eight bacteriophages (PT6, PT8, PNM, 14/1, 9/3, F77, PL and ISP) were selected for further matching against 23 *P. aeruginosa* and 17 *S. aureus* strains, recently isolated from patients at the Burn Centre of the Queen Astrid Military Hospital in Brussels. The data on strains and phages tested are shown in **Table S2**. This resulted in the selection of three bacteriophages that exhibited a large host range activity, specific for the burn wound isolates: PNM, 14/1 and ISP (**Table 1**).

**Table 1:** Characteristics of the three lytic bacteriophages present in BFC-1.

Phage	14/1	PNM	ISP
**Host species**	*P. aeruginosa*	*P. aeruginosa*	*S. aureus*
**Initial source**	Sewage water	Mtkvari River	Unknown
**Initial place of isolation**	Regensburg, Germany	Tbilisi, Georgia	Tbilisi, Georgia
**Initial date of isolation**	2000	1999	1920–1930
**Isolated by**	V. Krylov (SIGSIM)	N. Lashki & M. Tediashvili (EIBMV)	from ISP^a^
**Serogroup**	E	PT5, PNC101	ISP
**Genome size (kb)**	66.1	42.4	120
**Family of ** ***Caudovirales***	*Myoviridae* A1	*Podoviridae* C1	*Myoviridae* A1
**Host range activity (%)**			
All strains	37	44	91
BWC strains	83	96	100

a ISP: Intravenous Staphylococcal Phage (ISP) preparation produced by Eliava IBMV. Phage ISP was isolated from this preparation in the 1970s.

*P. aeruginosa* bacteriophage PNM, a member of the *Podoviridae* family, was isolated in 1999 from the Mtkvari river in Tbilisi, *P. aeruginosa* bacteriophage 14/1, a member of the *Myoviridae* family, was isolated by Victor Krylov in 2000 from sewage water in Regensburg, Germany and *S. aureus* bacteriophage ISP, also a member of the *Myoviridae*, was isolated from the Intravenous Staphylococcal Phage (ISP) preparation produced by the EIBMV in the 1970s. ISP was initially isolated in the 1920s from an unknown source in Tbilisi, Georgia.

Quality control parameters verified for this cocktail included sterility, pyrogenicity and pH stability, evaluated as described in the materials and methods section and further specified in **Table 2**.

**Table 2:** Summary of the quality control test results.

Test	Specifications	Method	BFC-1 test result
pH	6.0–8.0	pH test (EP6)	7.0 **(conform)**
Pyrogenicity	≤1.15°C temperature rise	Intravenous injection in 3 rabbits (EP6)	0.5°C **(conform)**
Sterility	Sterile	Membrane filtration method (EP6)	Sterile **(conform)**
Cytotoxicity	No cytotoxicity, no growth inhibition, no morphology changes	Co-culture with human keratinocytes	No cytotoxicity **(conform)**, **n**o growth inhibition **(conform)**, no morphology changes **(conform)**
Activity (titre)	log(8)–log(10) pfu/ml	Bacteriophage titration	8 log(8)–log(9) pfu/ml **(conform)**
Morphology of the bacteriophages	2 *Myoviridae* and 1 *Podoviridae*	Transmission electron microscopy	2 *Myoviridae* and 1 *Podoviridae* **(conform)**
Recognition of targeted bacteria	Specific recognition	Transmission electron microscopy	One *Myo*- and one *Podoviridae* member recognize and kill *P. aeruginosa*. The other *Myoviridae* member recognizes and kills *S. aureus* **(conform)**
Phage intactness Absence of cellular debris	Intact, pure	Transmission electron microscopy	Pure **(conform)**
Temperate bacteriophages in host strains	Total absence	Mitomycin C induction of temperate bacteriophages	Total absence **(conform)**
Lytic nature	Lytic	DNA sequence and proteome analysis	Lytic **(conform)**
Presence of toxic proteins	No toxic proteins	DNA sequence and proteome analysis	No predicted toxic proteins **(conform)**

### Transmission electron microscopy

**Figure 4:**
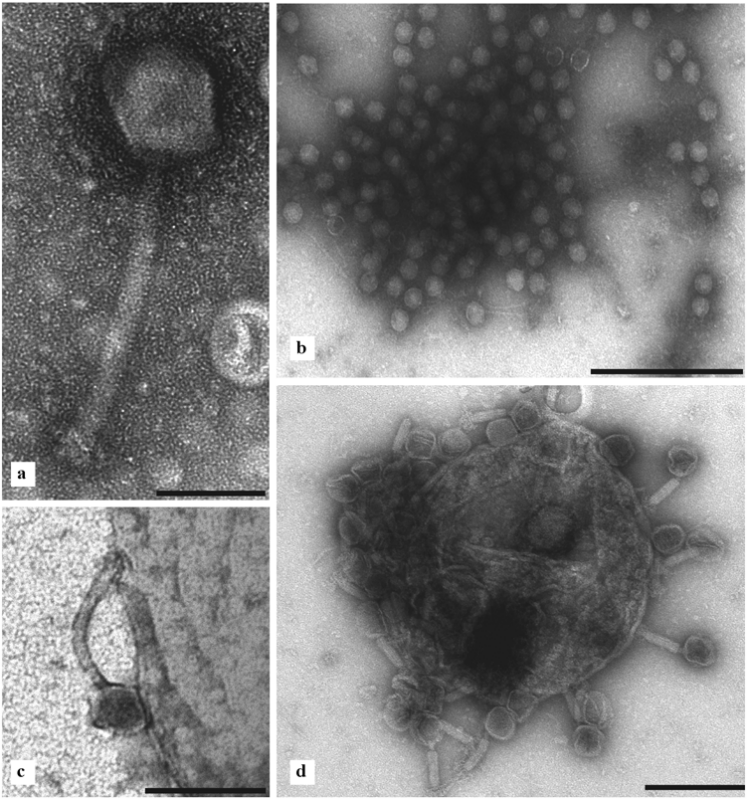
BFC-1 transmission electron micrographs. a) *S. aureus* bacteriophage ISP, a member of the *Myoviridae* family. Bar: 100 nm. b) PNM bacteriophages (*Podoviridae*) freed from a burst *P. aeruginosa* bacterium. Bar: 500 nm. c) Bacteriophage 14/1 attaching to the *P. aeruginosa* cell wall. Bar: 200 nm. d) ISP bacteriophages (*Myoviridae*) attached to *S. aureus*. Bar: 500 nm.

Microscopy confirmed that BFC-1 only contained bacteriophage particles with the expected morphology (**Figure 4**) and the expected target strain activity.

Different types of particles were observed consisting of a non-enveloped head with icosahedral symmetry and a tail with helical symmetry. These characteristics are consistent with the attributes of the bacteriophage order *Caudovirales*.

One type of particles had an isometric hexagonal head with a diameter of 60 nm and a straight, short, thick, non-contractile tail built of stacked non-banded rings. The tail had a length of 10 nm and a width of approximately 8 nm. Subterminal fibers were short and difficult to visualize. These characteristics attribute this type of bacteriophages to the *Podoviridae* family.

Two morphotypes of *Myoviridae* could be distinguished, based on the respective sizes of the head and the length of the (non-contracted) tail. One population had a head diameter of approximately 78 nm and a tail length of 100 nm, whereas a second morphotype had a head diameter of approximately 90 nm and a tail length of approximately 175 nm.

When BFC-1 was combined with cultured *S. aureus* and *P. aeruginosa* bacteria, bacteriophages from the *Podoviridae* and the *Myoviridae* adsorbed to (and killed) their respective host.

The material observed in BFC-1 was pure, consisting almost entirely of (exculsively lytic) bacteriophages, or bacteriophage-derived material (isolated tails, heads, etc). No other etiological agents or residual bacteria were observed.

### DNA sequencing and *in silico* proteomic analysis

The complete genome sequences of the two *P. Aeruginosa* infecting phages 14/1 and PNM were determined by a combination of shotgun sequencing and primer walking as described in Experimental Procedures. The genome of 14/1 comprises 66.2 kb and has a G+C content of 55.6%, which is significantly lower than that of its host (66.6%).

Sequence analysis showed that bacteriophage 14/1 is a close relative of bacteriophage F8 (66 kb) [31], having an overall DNA identity of 87% (total average), spread throughout the genome. Bacteriophage F8 is one of the original Lindberg *Pseudomonas* typing phages. Among the 90 predicted gene products of 14/1 (or corresponding homologsin F8 or F8-like bacteriophages like BcepF1 or BcepB1A) no toxic proteins are present and the genome does not contain a recognizable integrase gene, corroborating the lytic nature of these bacteriophages. Indeed, an initial screen revealed a single, potentially toxic gene (VirE) within the genome of phage 14/1 (NC_011703). However, the BCep1 VirE homologue was recently connected to the prim-pol primase superfamily of DNA polymerases, implying a role in phage DNA replication for VirE, rather than promoting host pathogenicity.

The genome of PNM is 42.7 kb and with its 62.3% it approximates the host G+C content.

The bacteriophage PNM genome shows close homology to φKMV, with only minor (single nucleotide) differences between PNM and φKMV, located in the DNA replication and structural region (average homology >95%). Differences are most obvious in the early region (80–90% DNA homology), which is consistent with the known genomic variations between the members of the φKMV-like bacteriophages [32]. To date, proteomic characterization of φKMV-like viruses has not revealed any toxic gene products present in these bacteriophages [32], [33], indicating their absence in PNM.

For bacteriophage ISP, the 816 sequencing runs on shotgun clones yielded a total of 120 kb of unique sequences, 100 kb of which is contained within contigs. This represents about 86% of the predicted genome length of about 140 kb. Preliminary analysis of the ISP genome data suggests that this bacteriophage is almost identical to *S. aureus* bacteriophage G1 (over 99% DNA homology). The homology of bacteriophage ISP to bacteriophages G1 and K is interesting from a therapeutic perspective, since these bacteriophages have been used in several clinical settings and animal studies [34], [35].

### Physicochemical properties of BFC-1

The pH of BFC-1 was 7.0, which is within specifications (6.0–8.0 pH).

Since ten percent of the BFC-1 production was found to be sterile as assessed by the membrane filtration method, the product was considered to conform to the European Pharmacopoeia standards (EP6).

The monthly titration of the separate bacteriophages in BFC-1 showed a conservation of 100% of the initial activity (1.10^9^ pfu/ml) for at least 12 months and further testing is ongoing.

### Toxicity and pyrogenicity of BFC-1

No cytotoxicity towards human neonatal foreskin keratinocytes was observed. The addition of BFC-1 to the culture medium had no impact on the viability of the keratinocytes (**Table 3**). The proliferation rate of the keratinocytes, represented by the mean cell number after 5 days of culture (**Table 3**), was not inhibited, and the morphology of the keratinocytes was not altered by the bacteriophages. Keratinocyte cultures, with and without BFC-1, reached 80–90% confluence after 5 days of culture whilst maintaining a normal morphology.

**Table 3:** Results of cytotoxicity testing.

Without BFC-1		
Culture flask	Cell number (log6)	Viability (%)
1	2.00	90.9
2	2.23	89.3
3	2.10	85.1
Mean:	2.11	**88.5**
Standard deviation:	1.17	3.0

With BFC-1		
4	1.90	93.4
5	2.30	90.8
6	2.33	89.7
Mean:	2.18	**91.3**
Standard deviation:	2.41	1.9

Since the sum of the increase of body temperature after injection of 1.2 ml of BFC-1 in the three rabbits was 0.5°C, which is much lower than the allowed increase of 1.15°C, the product was found to conform to the European Pharmacopoeia standards (EP6).

**Table 2** summarizes the tests, specifications, methods and results of the final quality control.

## Discussion

Phage therapy has the potential to be one of the promising alternatives/complements to antibiotics. In the past, this approach was often not as effective as hoped. Reasons for this included the empirical use of poorly characterised crude bacteriophage preparations. In addition, the clinical application of bacteriophages for treatment of infections of humans in modern western medicine is stuck in a vicious regulatory circle [14]. Under the current regulatory framework, bacteriophages do not exist because of the lack of clinical trials - yet to perform these trials one needs a regulatory existence. As such, the development of a well-characterized bacteriophage preparation using GMP (Good Manufacturing Practices)-like procedures was warranted for scientific and medico-legal reasons.

All products used in the production of BFC-1, were certified or accompanied by an adequate certificate of analysis and were fully compatible with the topical application on burn wound patients. In addition, all equipment (e.g. pipettes, bench flow and incubators) used in the production of BFC-1 was calibrated and certified. Apart from the bacteriophages (PNM, 14/1 and ISP), BFC-1 is composed of sterile and apyrogenic water (*aqua ad injectabilia* as required by the European Pharmacopoeia) as solvent, supplemented with 0.9% w/w NaCl for adaptation to physiologic osmotic strength.

The minimal bacteriophage titres required for the intended clinical applications are unknown. Bacterial loads of 10^5^ bacteria per g wound tissue were shown to confer a septicaemic risk [36]. Our choice of 10^9^ pfu/ml of each bacteriophage, applied in doses of 1 ml per 50 cm^2^ wound bed, should result in concentrations of at least 100 bacteriophages for each target bacterium.

Since it cannot be excluded that BFC-1 also contains traces of the initial bacterial growth medium and since traditional growth media for bacteria contain animal extracts (implying a risk of transmission of infectious agents such as BSE), it was decided to use a bacterial growth medium certified to be free of animal proteins. The most important medium remnants in BFC-1 are soy hydrolysate and yeast extract, inherent components of the Select APS LB Broth Base used for bacterial growth and bacteriophage production. The theoretical final concentrations of these remnants, before endotoxin removal, are 25 and 125 µg/ml of BFC-1 respectively.

Phage therapy faced several problems often due to an inadequate preparation methodology. Purification, removal of endotoxins and pyrogenic substances, stability and pH control of the preparation were rather problematic in the past [6]. Endotoxins possess a high degree of toxicity, and their removal is essential for safety in antibacterial bacteriophage therapy [37]. BFC-1 was easily and successfully purified from endotoxins using a commercially available, column endotoxin purification kit, which is based on the principles of affinity chromatography. The high endotoxin affinity ligand of the EndoTrap® Blue affinity matrix is proteinaceous and derived from a bacteriophage. It is not an antibody, and is covalently immobilized on agarose beads in order to ensure negligible leakage.

Accredited laboratories, able to deliver certified results, performed the final quality control tests such as determination of pH, pyrogenicity and sterility, classically required in clinical studies. An in-house cytotoxicity test was performed. For endotoxin testing, the *Limulus* Amoebocyte Lysate (LAL) assay is the regulatory first line test (EP6, chapter 2.6.14/11.2). The reference laboratory to which the cocktail was sent for pyrogenicity testing first attempted to apply the LAL assay, but the phages appeared to interfere with this test. The rabbit pyrogenicity test can be used only when there is interference with the endotoxin test, which was the case here. In fact the pyrogenicity test encompasses all pyrogens and not only endotoxins. We applied the real volumes that were to be applied on patients and took the largest safety factor for acceptability of the phage cocktail.

In theory, there is no need to ascertain the absence of pyrogens from products, which are not intravenously/parenterally administered. However, we worked with a product that during its production process was in close contact with bacteria and that by application to a burn wound could diffuse partially into the blood stream.

The pH is a homeostasis and stability indicator and thus an important parameter of a therapeutic product. A pH between 6.0 and 8.0 guarantees the stability of infectivity of the bacteriophages [38] and is compatible with the wound bed physiology [39]. The pH 7.0 of BFC-1 (ideal for stable storage) is suitable for topical use on burn wounds.

According to guideline Q5C (Quality of Biotechnological Products: Stability Testing of Biotechnological/Biological Products), published by the International Conference on Harmonisation of Technical Requirements for Registration of Pharmaceuticals for Human Use (ICH), the manufacturer should propose a stability indicating profile to ensure that changes in the identity, purity and potency of the product will be detected. Since BFC-1 consists of three bacteriophages suspended in a physiological solution and is stored at 2–8°C in 3 ml ‘single use only’ vials, identity and purity are not considered likely to alter. In this particular case, the parameter that is thus proposed to profile the stability characteristics of BFC-1 is the activity or potency of the sole active components of BFC-1, the bacteriophages PNM, 14/1 and ISP. The slightest deterioration of one or all of the bacteriophages will immediately result in a decrease of the capacity to achieve its intended effect ( = activity or potency) of BFC-1. This capacity was measured through titration of the bacteriophages against the host strains that were used to propagate the phages. BFC-1 was shown to maintain 100% of its initial activity after storage at 4°C for at least one year. Further follow-up of stability testing is ongoing.

Since temperate bacteriophages are known to transfer antibiotic resistance and virulence genes from one bacterium to another, a process known as lysogenic conversion, the absence of temperate bacteriophages from the host bacteria used in the production of BFC-1 had to be confirmed. The chemical induction test suggested an absence of temperate bacteriophages and the DNA sequence and proteomic analysis confirmed the lytic character of the bacteriophages and the absence of toxin genes. Transmission electron microscopy confirmed the phage particle intactness and expected morphology as well as the absence of cellular debris.

The host specificity of the phages could be established by means of electron microscopy as well.

Data documenting the BFC-1 production process and the certified quality control tests on the final product were compiled into a batch record file. An industrial pharmacist certified the conformity of this file to the product information file.

BFC-1 is currently evaluated in a clinical trial, which was approved by the Ethical Committee of the “Universitair Ziekenhuis” of the “Vrije Universiteit Brussel” and started in October 2007. To date, BFC-1 has been applied topically (**Figure 3**) on the infected burn wounds of eight patients. No adverse events were observed.

This is, to our knowledge, the first detailed description of a quality-controlled small-scale production of a bacteriophage preparation, leading to a safety trial in burn wound patients, which was approved by a leading Belgian ethical committee. The aim of this manuscript was therefore not to produce a commercial product, or to assess regulatory aspects of phage therapy, but merely implementing a small step in the further evaluation of phage therapy in Western medicine.

Together with European experts, we are currently in the process of creating a discussion platform that could act as one of the interlocutors with the regulatory authorities (e.g. EMEA) for the creation of a specific regulatory framework and appropriate production standards for phage therapy. Another, equally important objective is to help provide the necessary studies to enable a coherent use of phage therapy.

## Supporting Information

Table S1 Activity of phages against 113 *P. aeruginosa* and 99 *S. aureus* strains, isolated from different clinical and environmental habitats across the world.
(0.25 MB XLS)

Table S2 Activity of phages against 23 *P. aeruginosa* and 17 *S. aureus* strains, recently isolated from patients at the Burn Centre of the Queen Astrid Military Hospital in Brussels.
(0.03 MB XLS)
